# Supplementary figures and images for: CHST4 Gene as a Potential Predictor of Clinical Outcome in Malignant Pleural Mesothelioma
Source: Int J Mol Sci. 2024 Feb 14;25(4):2270. doi: 10.3390/ijms25042270 (PMC10889779; doi:10.3390/ijms25042270)

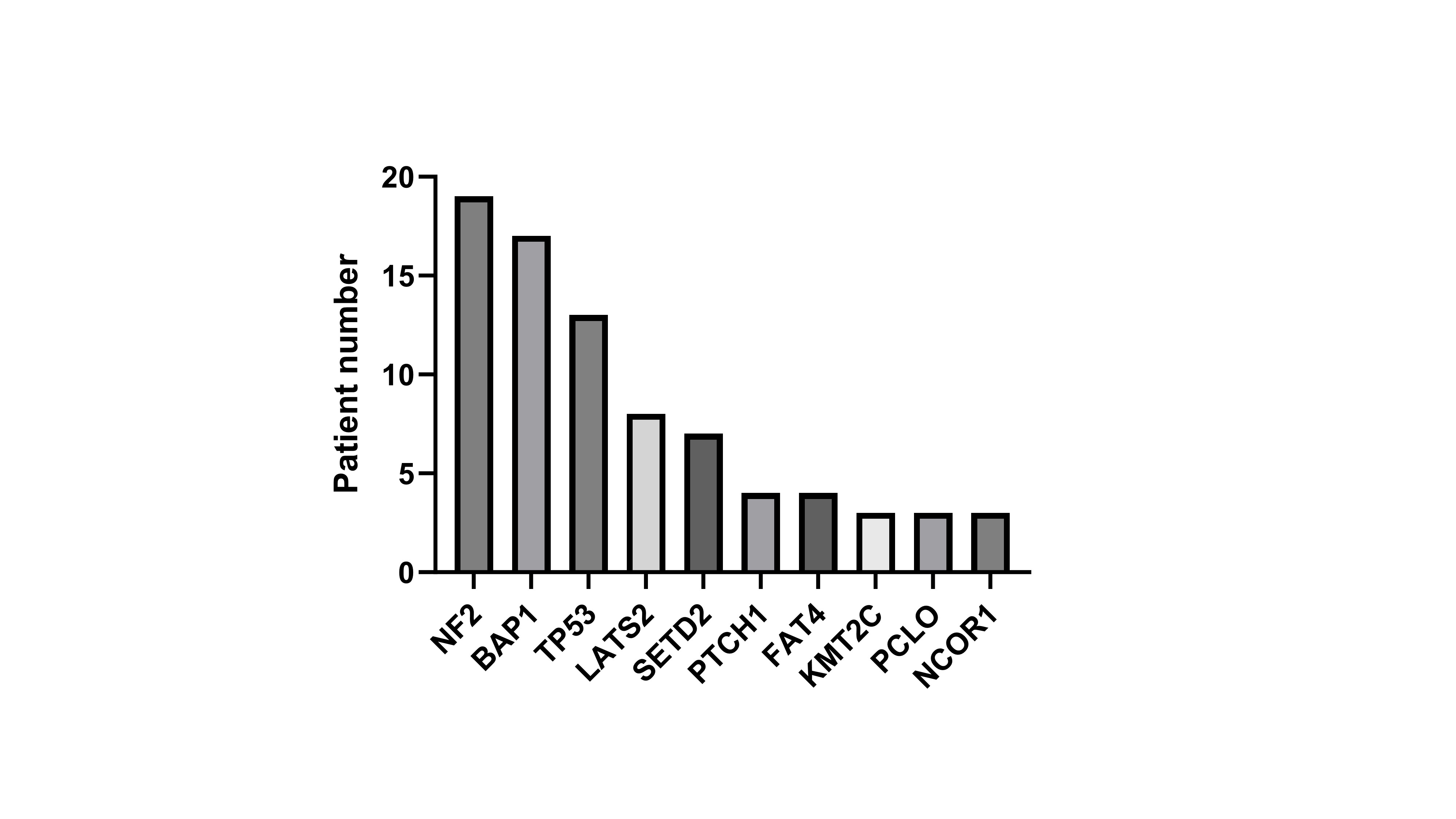

Supplement: Supplementary file 1 [file ijms-25-02270-s001.zip › Figure S1.jpg]

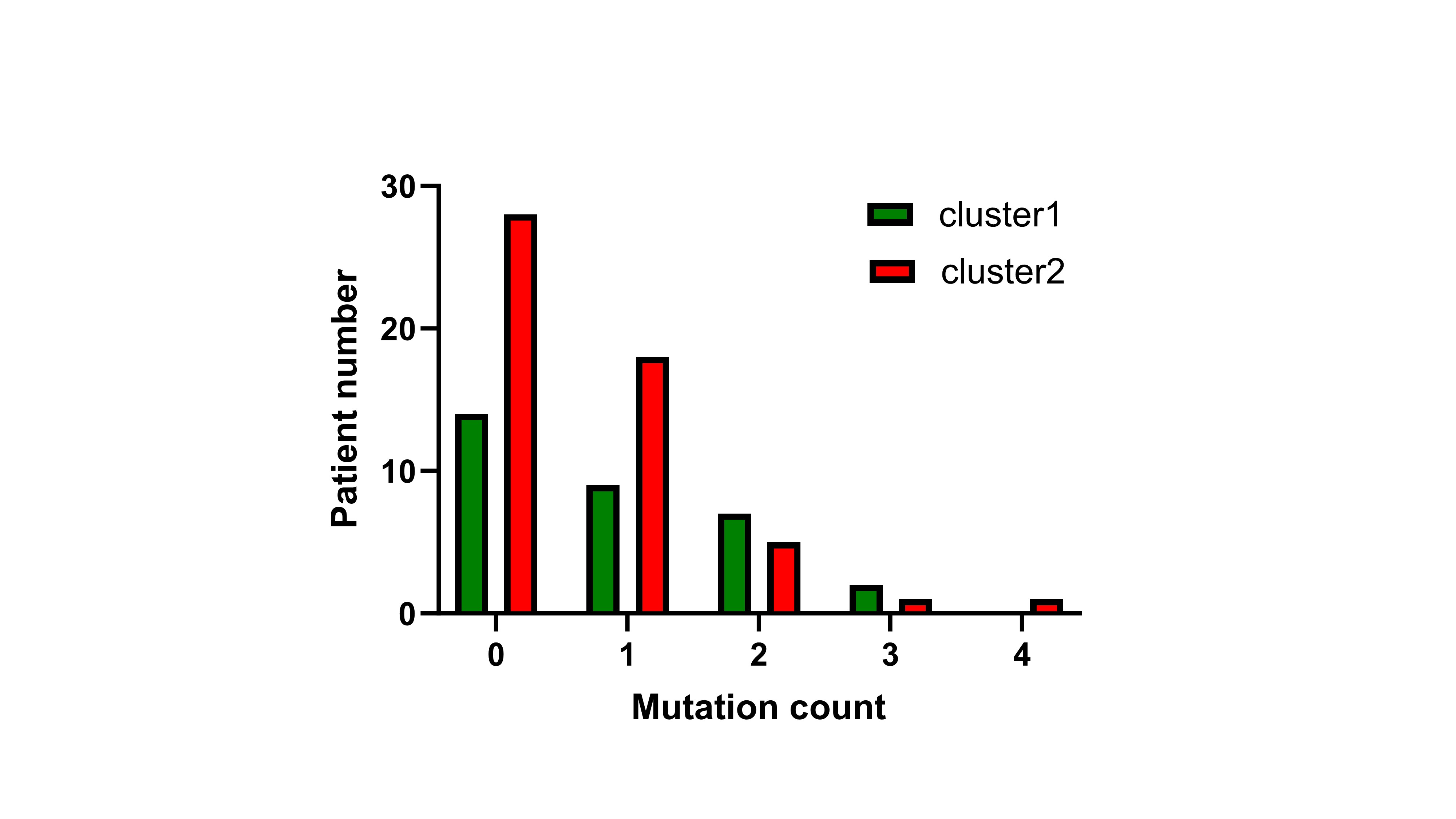

Supplement: Supplementary file 1 [file ijms-25-02270-s001.zip › Figure S2.jpg]

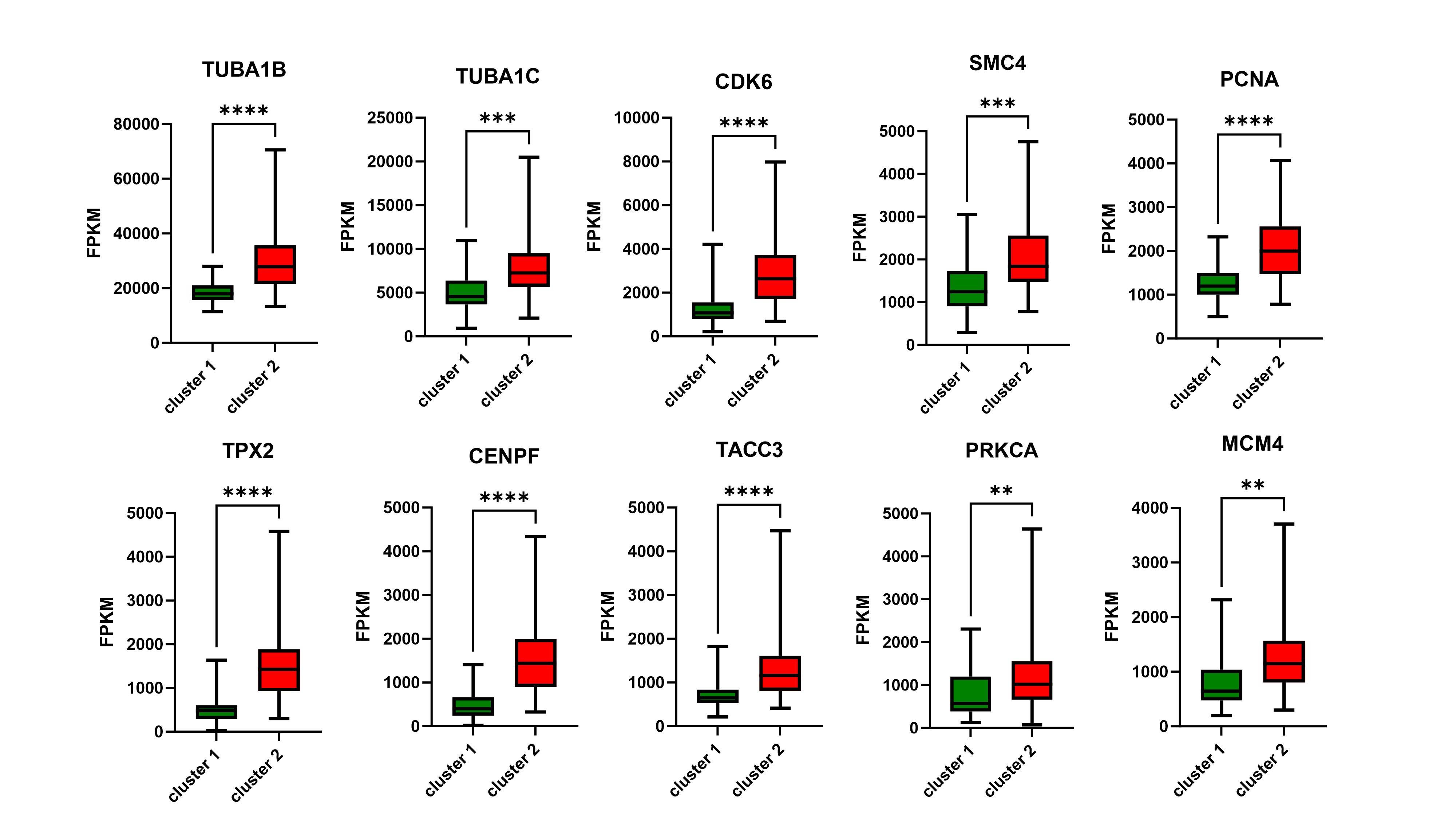

Supplement: Supplementary file 1 [file ijms-25-02270-s001.zip › Figure S3.jpg]

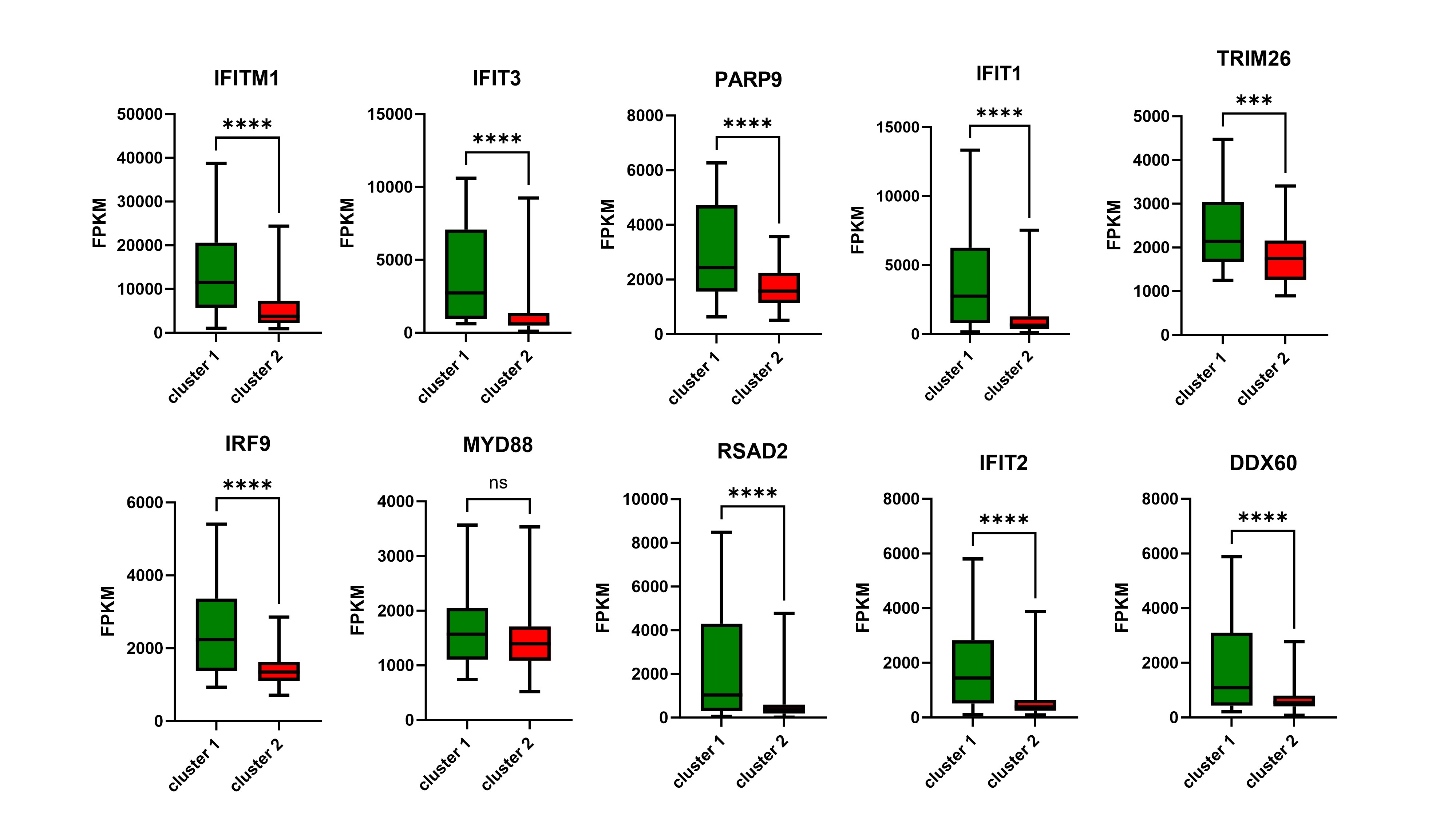

Supplement: Supplementary file 1 [file ijms-25-02270-s001.zip › Figure S4.jpg]

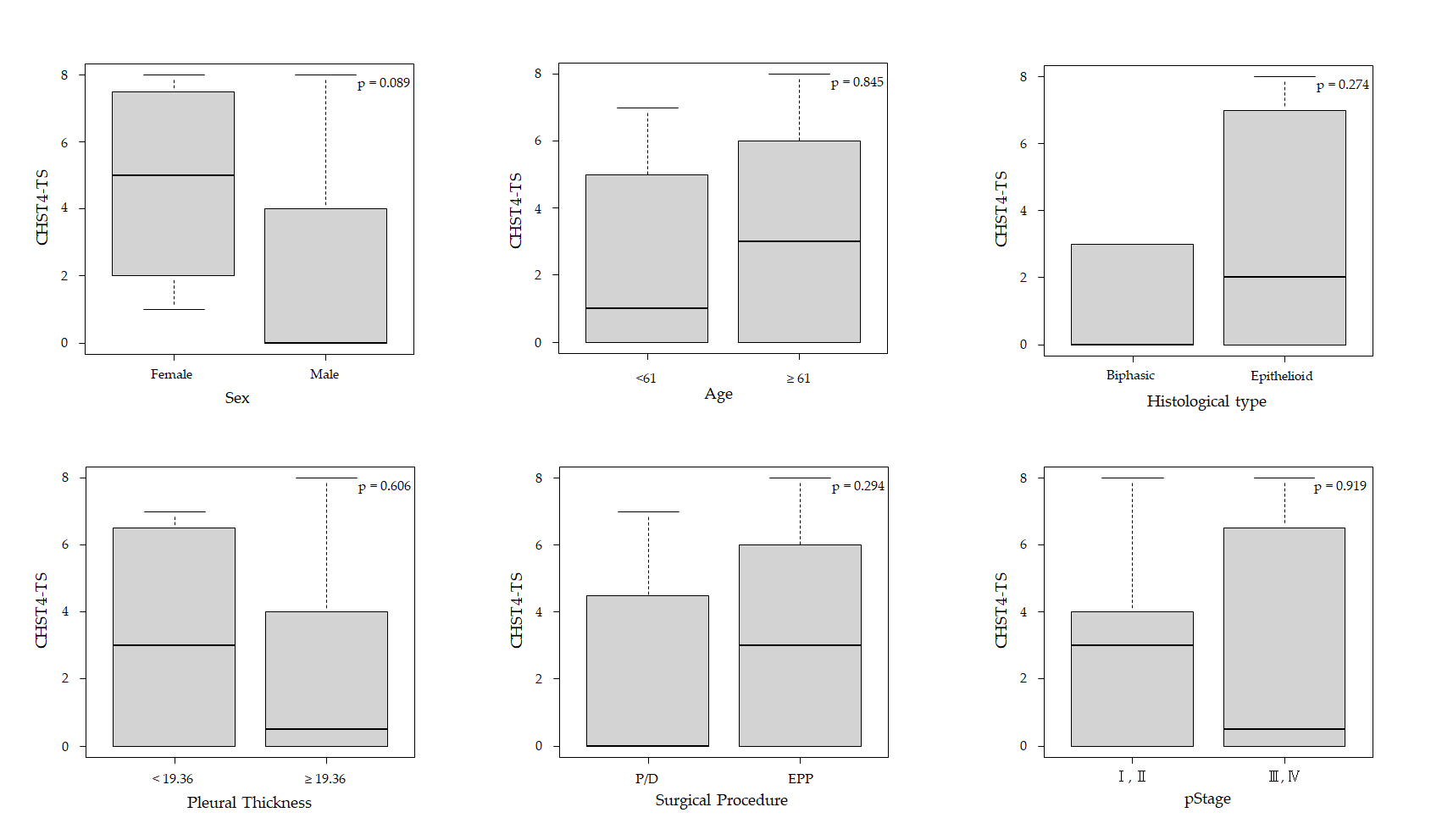

Supplement: Supplementary file 1 [file ijms-25-02270-s001.zip › Figure S5.png]

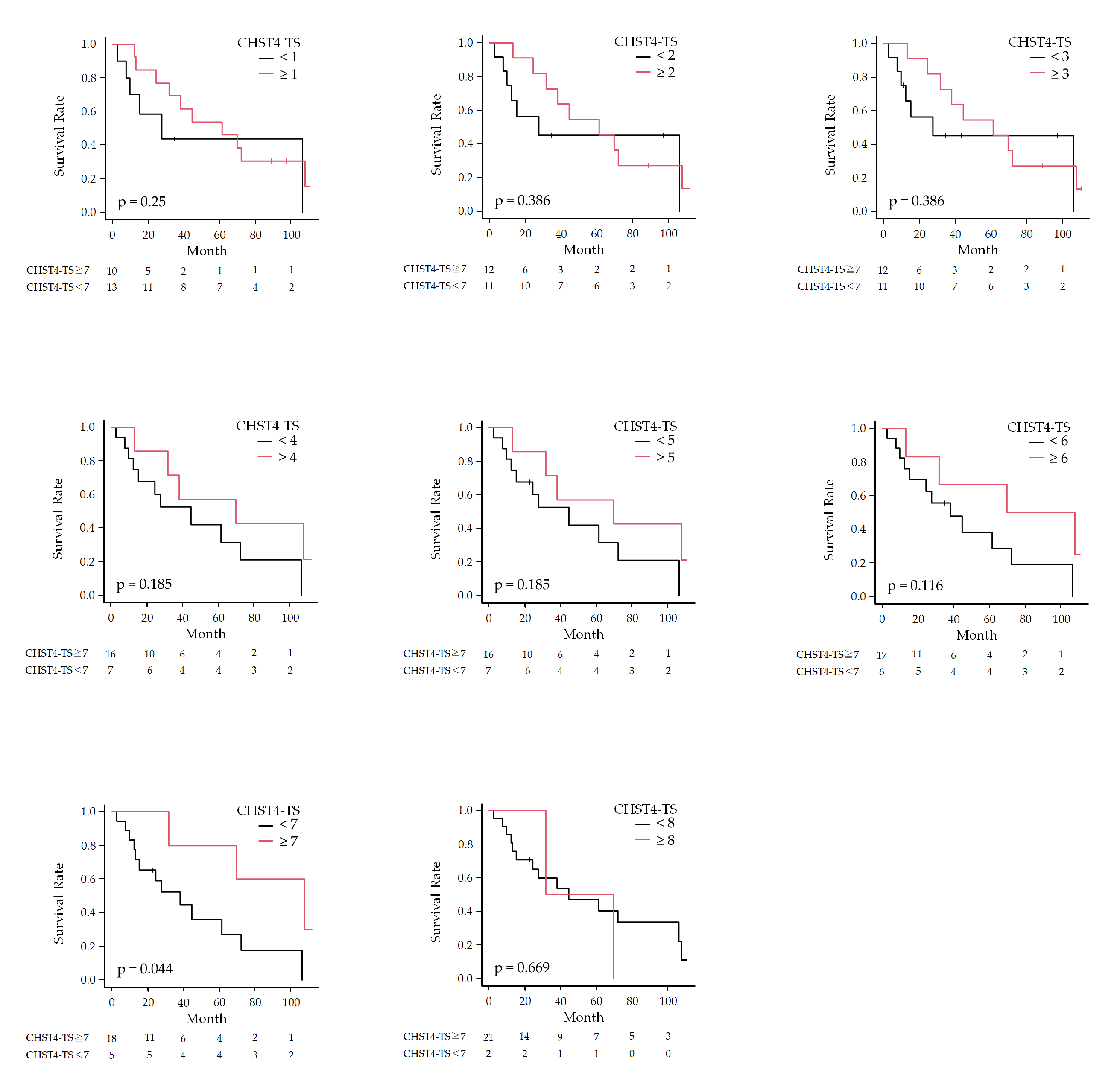

Supplement: Supplementary file 1 [file ijms-25-02270-s001.zip › Figure S6.png]

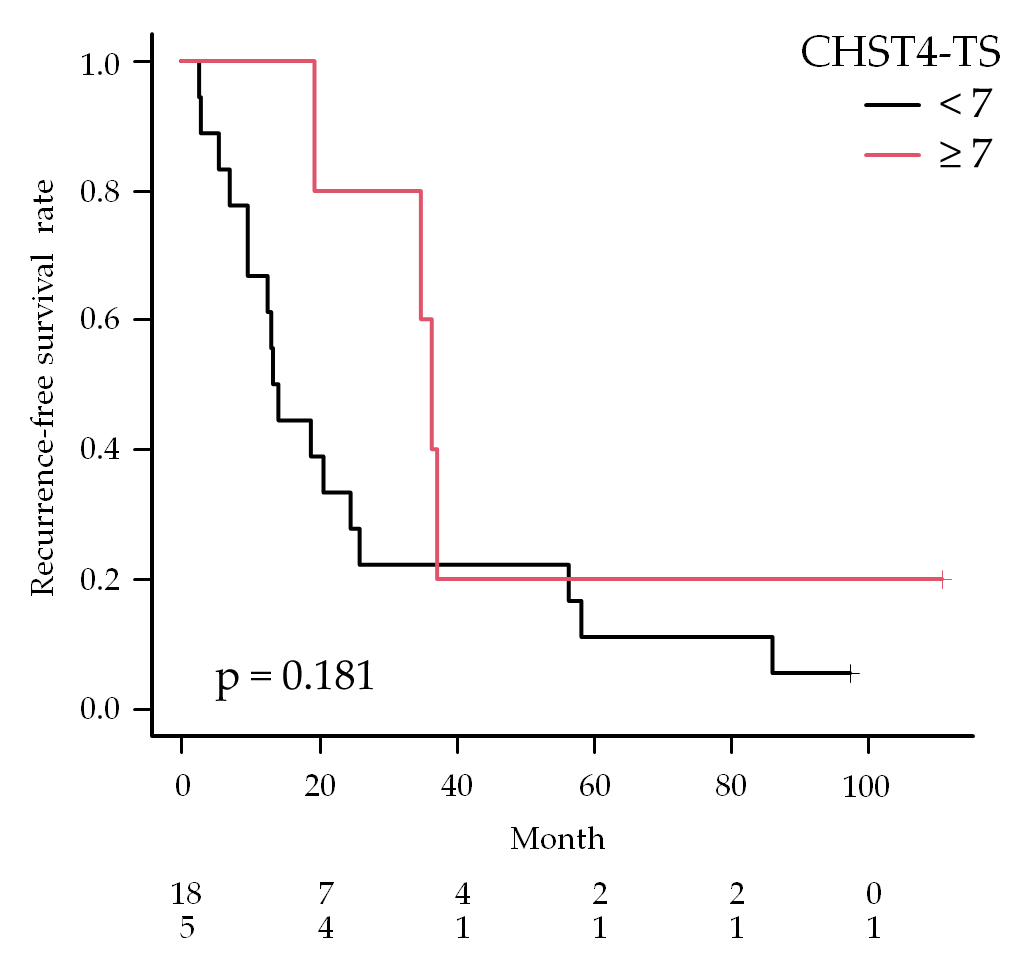

Supplement: Supplementary file 1 [file ijms-25-02270-s001.zip › Figure S7.png]
